# Anterior laryngeal membrane and 22q11 deletion syndrome

**DOI:** 10.1590/S1808-86942011000400024

**Published:** 2015-10-19

**Authors:** Rafael Fabiano Machado Rosa, Rosana Cardoso Manique Rosa, Rita Carolina Pozzer Krumenauer, Marileila Varella-Garcia, Giorgio Adriano Paskulin

**Affiliations:** 1 Master's degree, medical geneticist at the UFCSPA/CHSCPA. Doctoral student in pathology (Graduate Program in Pathology), UFCSPA; 2 Specialist in pediatrics, master's degree student in pathology (Graduate Program in Pathology), UFCSPA. Brazil; 3 Master's degree in health sciences, otorhinolaryngologist, tutor at the Otorhinolaryngology Unit, HCSA/CHSCPA, Brazil; 4 Doctoral degree in biological science, professor and cytogeneticist in charge of the Cytogenetics Laboratory, Medical Oncology Division, University of Colorado Denver, US; 5 Doctoral degree in genetics and molecular biology, medical geneticist at UFCSPA/CHSCPA. Professor in the Clinical Genetics Discipline and the Graduate Program on Pathology, UFCSPA. Cytogeneticist in charge of the Cytogenetics Laboratory of the UFCSPA, Brazil

**Keywords:** chromosomes, human, pair 22, DiGeorge syndrome, in situ hybridization, fluorescence, larynx

## INTRODUCTION

Anterior laryngeal webs (ALWs) are uncommon abnormalities consisting of membranous tissues on the supraglottic, glottis, and/or subglottis at birth.^1^, ^2^, ^3^ These webs answer for about 5% of laryngeal malformations;^1^ depending on how extensive they are, airway obstruction may ensue, resulting in symptoms such as crying, stridor, dysphonia, and respiratory dysfunction.^2,^^4^ Individuals with this condition often present other concomitant anomalies, such as congenital heart defects, and palatine anomalies, which often are part of known genetic syndromes.^1^, ^2^, ^3^

This paper presents a case report of a patient with an ALW and the 22q11 deletion syndrome (SD22q11), also known as the velocardiofacial syndrome or DiGeorge syndrome (*OMIM* 188400/192430).^5^

## CASE REPORT

A male Caucasian patient aged 12 years and 2 months was first admitted to hospital for surgery to correct a fossa ovalis type interatrial communication. The patient was the first child of young, healthy, and non-consanguineous parents. The family history was negative for congenital defects or genetic diseases. Pregnancy coursed uneventfully. The patient was delivered vaginally, at term, cephalic presentation, weighing 3,430 gr. (P50), measuring 50 cm (P25-50), with a 35 mm cephalic perimeter (P50-98). The baby was cyanotic and did not cry during birth. Oxygen therapy was required, and the patient remained in hospital for 15 days after birth. At the age of 3 months, a laser laryngeal procedure was done to remove a subglottic membrane; at this point the patient had episodes of hypocalcemia that required treatment with calcium gluconate.

**Figure 1:**
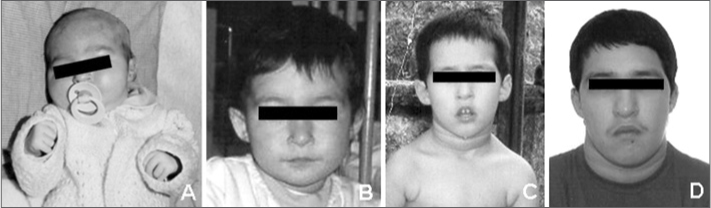
Craniofacial features of the patient at different ages: 16 days (A), 1 year and 11 months (B), 4 years (C), and 13 years (D).

Neuropsychomotor, behavioral, and speech development was compromised as the patient grew, requiring drug therapy - haloperidol and biperiden. The physical examination at age 12 years and 2 months was as follows: weight - 47 kg (P75-90), height - 147 cm (P50), cephalic perimeter - 53 cm (P2-50), elongated face, narrow palpebral fissures, bilateral epicanthic folds, hypoplasic nasal alae, dental malocclusion, prognathism, overfolded helices (horizontal and vertical rami), *pectus carinatum*, protruding navel, right cryptorchidism, and thin fingers of the hand. The palate was ogival, but there were no associated abnormalities such as velopharyngeal insufficiency. Figure 1 shows the craniofacial features of the patient at different ages. The patient was referred to an otorhinolaryngologist due to dysphonia of unknown origin. Nasofibrolaryngoscopy revealed the presence of an ALW associated with anterior subglottic stenosis and decreased glottic lumen. The trachea and the carina were normal. The child did not present dyspnea. At this point the decision was in favor of watchful waiting. Abdominal ultrasound revealed only an accessory spleen. The blood calcium level was within normal limits. The initial cytogenetic evaluation - high resolution GTG band karyotyping (≥ 550 bands) - was normal (46, XY). Investigation of the 22q11.2 microdeletion with the fluorescent in situ hybridization technique (FISH), using a DNA DiGeorge/VCFS Region Probe (TUPLE 1) DNA probe, confirmed the diagnosis of the SD22q11.

## DISCUSSION

ALWs are a mild form of laryngeal atresia (type III).^4^ Its association with the SD22q11 microdeletion has been described in a few published papers within the past two decades; most of these reports had a small number of patients.^1^, ^2^, ^3^, ^4^ Miyamoto et al. (2004)^3^ reported a 65% frequency of the SD22q11 in 17 patients with ALWs, one of the few case series. Studies of patients with the SD22q11 have noted that this malformation has been described in 1 to 2% of cases.^6^

The SD22q11 is a relatively common genetic disease; it is caused by a deficiency in region 11 in the long arm of the chromosome 22. Although most cases of the SD22q11 are sporadic (because of new mutations), patients with this deletion have a 50% chance of passing it onto their offspring. The clinical features of this syndrome are varied; the phenotype includes several types of otorhinolaryngeal alterations. These changes have been given several names given before the syndrome was described in the beginning of the 1990s, such as the DiGeorge syndrome, the velocardiofacial syndrome, and Shprintzen's syndrome. These names reflect the perception of different specialists about the same disease.^6^

It should be noted that in some cases these features may not be so evident, especially younger children, making it more difficult to raise the possibility of this syndrome^3,^^5^ (see Fig. 1). Hypocalcemia may be latent, as in this case, and may become apparent only before surgery.^7^

## COMMENTS

As has been reported in the literature,^1^, ^2^, ^3^, ^4^ these findings suggest that patients with ALWs - and particularly those with other congenital malformations such as heart defects - should always be tested for the SD22q11 deletion syndrome. This has important implications for the management of patients and genetic counseling of the family.
